# The integrated GOR-COVID-19 health monitor: protocol for a comprehensive approach: Anouk van Duinkerken

**DOI:** 10.1093/eurpub/ckac129.479

**Published:** 2022-10-25

**Authors:** A van Duinkerken, M Bosmans, N Tak, C Baliatsas, N Jansen, M de Vetten-Mc Mahon, E Marra, M Dückers

**Affiliations:** 1 Disasters and Environmental Hazards, NIVEL, Utrecht, Netherlands; 2 GGD GHOR Netherlands, Utrecht, Netherlands; 3 Impact, ARQ National Psychotrauma Centre, Diemen, Netherlands; 4 Centre for Environmental Safety and Security, RIVM, Bilthoven, Netherlands; 5 Faculty of Behavioural and Social Sciences, University of Groningen, Groningen, Netherlands

## Abstract

**Introduction:**

The global COVID-19-pandemic influences people's health, both directly through infection and indirectly through the protective measures taken by governments. Previous experience with health research after disasters/crises are generally limited to short-lasting, local disasters with direct consequences for those affected. The COVID-19-pandemic has a different nature: influencing everyone and lasting a longer time. A longitudinal, wide-reaching research-approach is needed to study the health effects of COVID-19. Therefore, the Network GOR-COVID-19, a research group consisting of different organizations, started a monitor on the health effects of COVID-19.

**Methods:**

The monitor consists of three main elements: yearly monitoring, quarterly monitoring and literature reviews. Where possible, existing data structures are used. For the quarterly monitoring, two data sources are used: general practitioners’ [GP] registry data and data gathered from panels. The GP data is used for weekly surveillance, giving insight into the prevalence of health symptoms presented to the GP. The panel data is used to gain insight into current self-reported health and wellbeing of people. For the yearly monitoring, two data sources are used. The first is GP data which gives information about the prevalence, incidence and development of symptoms, complaints and diagnoses. It allows for comparison over time and among different population groups. The second is the corona health monitor questionnaire, an existing questionnaire on health and well-being. Finally, literature reviews are conducted annually to create an overview of international and national research about the effects of the COVID-19-pandemic on health.

**Discussion:**

Since most of our knowledge about the potential impact of the pandemic stems from research on short-term disasters, limited to specific places, this study is expected to provide valuable new insights.

